# Chronic Disease, the Built Environment, and Unequal Health Risks in the 500 Largest U.S. Cities

**DOI:** 10.3390/ijerph17082961

**Published:** 2020-04-24

**Authors:** Kevin M. Fitzpatrick, Don Willis

**Affiliations:** 1 Department of Sociology and Criminology, University of Arkansas-Fayetteville, Fayetteville, AR 72701, USA; 2 Department of Sociology and Anthropology, University of Arkansas-Little Rock, Little Rock, AR 72204, USA; dewillis@ualr.edu

**Keywords:** chronic disease, place and health, cardiometabolic disease, structural inequality

## Abstract

Health is increasingly subject to the complex interplay between the built environment, population composition, and the structured inequity in access to health-related resources across communities. The primary objective of this paper was to examine cardiometabolic disease (diabetes, cardiovascular diseases, stroke) markers and their prevalence across relatively small geographic units in the 500 largest cities in the United States. Using data from the American Community Survey and the 500 Cities Project, the current study examined cardiometabolic diseases across 27,000+ census tracts in the 500 largest cities in the United States. Earlier works clearly show cardiometabolic diseases are not randomly distributed across the geography of the U.S., but rather concentrated primarily in Southern and Eastern regions of the U.S. Our results confirm that chronic disease is correlated with social and built environment factors. Specifically, racial concentration (%, Black), age concentration (% 65+), housing stock age, median home value, structural inequality (Gini index), and weight status (% overweight/obese) were consistent correlates (*p* < 0.01) of cardiometabolic diseases in the sample of census tracts. The paper examines policy-related features of the built and social environment and how they might play a role in shaping the health and well-being of America’s metropolises.

## 1. Introduction

Health is increasingly subject to the complex interplay between the built environment and the pervasive structural and racial inequality across communities in the United States. Social and economic deprivation whether in the form of inadequate housing, limited access to healthy foods, social isolation, heightened levels of violence, or other forms of social disconnectedness, continue to provide a more complete understanding of health disparities in America [1,2,3,4,5]. Of particular concern has been the recent uptick in chronic illness prevalence linked to lifestyle, diet, and preventable negative behaviors. In 2012, over 700,000 U.S. deaths were attributed to cardiometabolic diseases such as heart disease, stroke, and type 2-diabetes [6,7]. Nearly half of those deaths were associated with poor diet [6]. This locates all three of these nutrition-related cardiometabolic diseases among the top ten leading causes of death in the U.S, as well as for early deaths, or years of life lost [8,9]. Mokdad and colleagues [9] estimated that dietary risks accounted for over 500,000 deaths in 2016. In a recent report, the Centers for Disease Control and Prevention (CDC) reported that the obesity rate in the U.S. has reached 42 percent, registering a more than 40 percent increase in the last two decades [10]. Clearly, food and diet are playing a major role in the prevalence of preventable chronic diseases in the United States.

Food and diet, however, exist in and are often shaped by space and place. The burden of disease, while driven largely by diet, is nevertheless unevenly spread across U.S. states [9,11,12]. Focusing on individual dietary risk factors alone neglects the fact that diets and behaviors are embedded in and shaped by larger social, cultural, and physical contexts (e.g., access to, acceptance, and affordability of healthy foods). Among the key social determinants of cardiovascular disease are social relationships, structural vulnerabilities, and geographic environment [13]—all of which are examined in the present study. The literature highlighting the intersection of health and place provides important preliminary evidence of the unequal distribution of chronic diseases across varying places [11,12,14,15]. Research demonstrates the distributional character of disease in geographically limited studies where disease and health risks are concentrated in poor, segregated, minority communities. However, it remains unclear how this distribution looks across all of the largest urban areas in the United States. With a growing recognition that context shapes choices, ecological models that highlight the interconnectedness of place, choices, and the environment’s impact on health and disease, continue to gain momentum across scientific disciplines [16,17,18].

In assessing health disparities across the United States, research highlights a number of critical social determinants, including structural vulnerabilities that are determined largely by the distribution of certain population subgroups across communities [1]. Race, age, ethnicity, and gender have long been noted as important determinants in explaining variation in disease and illness. Health chances are often a function of who you are and where you live and an extant literature has documented the important intersection of this vulnerability and its relationship to place [19,20,21]. While access to health care resources remains an important correlate of explaining disease outcomes, social, cultural, political, residential, and economic factors continue to play an even greater role in understanding the geographic distribution of disease prevalence [22].

Additionally, characteristics of the built environment along with the socioeconomic composition of communities continues to tell a key part of the story about who is at a higher risk and why when it comes to chronic disease. Socioeconomic disadvantage, which is typically found in deprived neighborhoods, is an important correlate of health disparities. While these are critical to explaining the wide variation in disease and illness across different groups and communities, a more complete and comprehensive picture of what all these factors are and how they impact varying disease prevalence is needed and is the focus of the current study.

Using data from the American Community Survey (ACS) [23] and the 500 Cities Project [24], the current study examines cardiometabolic diseases that are diet- and nutrition-related across 27,000+ census tracts located in the 500 largest cities in the United States. Structural vulnerabilities, housing stock characteristics, and social inequalities are examined as important systemic correlates of chronic disease prevalence across these places; specific elements of both the economic structure and the built environment (i.e., inequality, housing, and food assistance) are examined as important place-based correlates of three chronic cardiometabolic disease outcomes. Using linear regression models, we examine individual coefficient effects, significance, and overall model fit among these selected variables. The residuals of each regression appear normal in both probability versus probability (P-P) plots as well as when plotted against predicted values. Finally, the paper explores the public health significance of these findings and their impact on statewide prevention and intervention programs related to diet, nutrition, and overall population health.

## 2. Data and Methods

The 500 Cities Project was designed to identify and analyze city- and census tract-level data using small area estimation for an examination of the geographic distribution of chronic diseases across the 500 largest American cities. The measures created from the 2015 Behavioral Risk Factor Surveillance System (BRFSS) data included unhealthy behaviors (e.g., current smoking), chronic disease prevalence (e.g., coronary heart disease, diabetes, etc.), and prevention behaviors (e.g., health insurance coverage, cholesterol screening, etc.). The BRFSS data is based on phone interviews conducted with U.S. adults each year. The BRFSS, sponsored by the Centers for Disease Control and Prevention (CDC), is the premier system of health-related telephone surveys that collect state data from U.S. residents regarding their health risks behaviors, chronic health conditions, and use of preventive services. BRFSS completes more than 400,000 adult interviews annually and the health data that are being used in this paper as the dependent variables were collected in 2015. BRFSS data collection started in 1984 and has been updated and refined over the last three decades, yet remains the gold standard for telephone interview survey data on health and health behaviors among adults living in the United States. With a response rate approaching 50 percent, measures are generally considered to be reliable and valid and have been examined in detail to confirm that conclusion [25].

Using these 500 Cities Project data, we compiled and merged them with the American Community Survey data from over 27,000 census tracts that were located in the 500 largest U.S. cities; 794 census tracts (2.8%) were eliminated from the analysis, because they contained fewer than 50 residents in the census tracts and thus any prevalence rates were redacted [24]. The final analysis uses data on 497 of the largest American cities and additional cities of Burlington, Vermont, Charleston, West Virginia, and Cheyenne, Wyoming, to ensure the inclusion of cities from all 50 states. The population of these largest cities ranges from 42,417 in Burlington, VT, to 8,175,133 in New York City, NY. The population of these census tracts ranges from less than 50 (removed from the current analysis) to 28,960 persons. The estimated total population that is included in the final analysis is more than one-third of the total U.S. population in 2010 (103,020,808). The analysis presented did not require any human subjects (Institutional Review Board) approval, because all personal identifiers were removed from these secondary data.

### Measurement

The primary objective of this paper was to examine cardiometabolic disease (diabetes, coronary heart disease, stroke) prevalence across relatively small geographic units in the 500 largest cities in the United States. As such, we examined prevalence (percentage) as estimated by the 2015 BRFSS among adults 18 years of age and older. For each of the three health outcomes used in this analysis, respondents 18 years of age and over confirmed that they were told by a health professional that they had heart disease, diabetes, or stroke. Using a multilevel regression and poststratification (MRP) approach, prevalence estimates are calculated at the census tract and city levels. This approach was employed prior to the release of the public data and readers can learn more about those details in methodology descriptions prepared by the 500 Cities Project [24].

Independent variables were obtained from the American Community Survey and other Bureau of Census Population Files [23,26]. These census tract level predictors (independent variables) are distinct from any of the contextual level measures that the 500 Cities project uses to estimate the prevalence of cardiometabolic diseases. Several measures were identified as important compositional correlates that included census tract-level population composition, census tract housing characteristics, and socioeconomic characteristics. Specifically, we include the following in the analysis: percentage of Black people, percentage of persons 65 years of age and over, the median home value, the median year housing structures were built, the Gini index of income inequality [27], the percentage of households with at least one person under the age of 18 that received Supplemental Nutrition Assistance Program (SNAP) benefits, the percentage of persons with less than a high school degree <25 years old, and a dichotomous measure that is an assessment of the overall health of the residents as it relates to cardiometabolic, chronic disease—a binary measure of normal vs. overweight/obese residents. The multivariate analysis focused on the statistical significance (*p* < 0.05) and confidence intervals (CI 95%) of individual unstandardized regression coefficients across each of the three disease models as well as a summary measure of the overall fit of the models (*R^2^*).

## 3. Results

While maps that have been generated by the 500 Cities Project clearly note important regional differences in the distribution of chronic metabolic diseases, we were interested in examining community-level correlates that are often discussed within the context of poor health outcomes—specifically, we were interested in whether there were any differences in chronic disease outcomes given certain population composition, housing, and socioeconomic characteristics across communities. Table 1 presents the descriptive statistics for both the independent and dependent variables used in the analysis. Descriptives show considerable differences in disease rates with over 30 percent reported for high blood pressure and a much lower prevalence rate of stroke found across these communities.

Table 2 presents results of linear regressions for three separate chronic diseases (type 2 diabetes, high blood pressure, and stroke) regressed on structural vulnerabilities (% Black and % elderly), housing stock (median home value and median year housing was built), socioeconomic composition (% with no high school education, % households receiving SNAP without any children under the age of 18, the Gini index of inequality), and weight status (% overweight and/or obese). Not surprisingly, all of these variables were significant (*p* < 0.001) across all three models except for the percentage without a high school education and its relationship with high blood pressure. Nevertheless, higher levels of income inequality and percentage of households receiving SNAP without children were positively associated with higher prevalence rates for all three chronic diseases. In addition, those census tracts with older housing stock and higher median home values were significant and negatively related to chronic disease rates. The *R^2^* was 0.50 for rates of high blood pressure, 0.54 for rates of stroke, and 0.54 for rates of diabetes, suggesting that these models explain roughly half of the variation in chronic disease prevalence in the 500 largest U.S. cities.

## 4. Discussion

Population composition, inequality, housing stock age and value, Gini index, SNAP participation, and weight status were consistent indicators of disadvantage across three chronic cardiometabolic diseases. Inequality operates negatively on human health through several pathways, but many studies suggest that it is a stressor due to the way in which it shapes our relationships, distribution of and access to resources, our sense of self, and our social positioning within relationships [27]. Only one of many indicators of the risk related to housing, the age of housing stock, captures a key characteristic of the built environment related to the basic need of shelter and protection against natural elements and chemical hazards. For example, homes built prior to 1978 still commonly contain lead-based paint [28,29], which can lead to contamination and cause serious health risks for residents if improperly renovated or repaired [30]. The prevalence of SNAP participation among families with children indicates material hardship in the form of individuals and families struggling to access affordable food. Given that dietary risks accounted for over 500,000 deaths in 2016 [9], access to and affordability of food is a serious factor in the social patterning of diet-related diseases like the ones we have examined in this study. Taken together, these three factors indicate widespread risk to human health through a variety of pathways.

In addition to sharing the characteristic relating to fundamental human needs, place-based variables such as inequality, housing, and SNAP participation also share another key characteristic: they can be shaped by human action, namely, public policy. As urban areas continue to grow—over 90 percent of Americans live in metropolitan regions [31]—large cities play an increasingly important role in the shaping of population health outcomes. Public policy at the city, regional, state, and national level could impact these contextual factors which are linked to cardiovascular disease. Progressive tax policies can be an effective means for flattening levels of inequality. Reasonable minimum wage laws can reduce the need for SNAP participation among families with children and hold companies accountable for providing living wages capable of feeding more than the individual worker. Some cities, such as Portland, have also put in place a policy aimed to reduce inequalities not just by raising the incomes of those at the bottom, but also by reining in the accumulation of income gains and wealth at the top—Piketty [32] has shown the primary driver of increasing inequality to be excessive returns on capital. This type of approach is particularly critical, as it recognizes the relational character of inequality wherein poverty and wealth accumulation are not separate but inherently linked phenomena.

While the focus on cardiometabolic diseases has been primarily located in the United States and other developed nations, developing nations are increasingly entering new phases of the epidemiological transition which place them at risk of these diseases as well [33]. Future research should include attention to the social and geographic patterning of these diseases in developing nations as they move through the epidemiological transition. Further, while there is some debate between those who highlight the importance of social relationships and others who emphasize objective material conditions [34,35], we see these as interactive rather than competing characteristics of the overall social environment and have included indicators of both in this study—we encourage future researchers to do so as well.

Finally, we recognize that this paper is not without limitations. There are many environmental and living condition variables beyond the scope of this paper which are also important to consider when analyzing the social and geographic patterning of chronic disease. The data are cross-sectional, which limits our ability to draw any causal conclusions. Nevertheless, any glimpse at the non-random, systematic, geographic distribution of chronic diseases and their related risks are an important first step toward continuing to argue about why place matters in understanding the health of America. In addition, the data/measures used in the present study to assess the social and built environments are limited by the kinds of measures that secondary sources like the U.S. Census provide at specific levels of context (tracts, blocks, etc.). We would encourage further and more detailed examinations of these risks at a variety of contextual levels of both place and health while asking additionally about the resources and protective factors that might help to moderate/mediate these risks on health outcomes.

## 5. Conclusions

Death and disease are not random, but socially produced [36,37,38]. The burden of cardiometabolic disease, a leading cause of death in the United States, follows social and geographic patterns—in particular, structural vulnerabilities, degrees of inequality, quality of housing stock, and access to benefits and/or the ability to afford food. While many of these relate to the material conditions of people’s lives, they are also closely linked to public policy decisions and social lives [39]. This study has demonstrated a strong link between place and diet-related diseases. We expected to find such a link, in part due to the strong connection between place and food [40], race [41], and inequality [39]. The connection between place and so many social determinants of health is due, in part, to the fact that “Space and place play a central role in organizing social life” [42]. As a fundamental organizing force for social life, it is frequently the characteristics of place, such as inequality, racial segregation, or material deprivation, that put populations at “risk of risk” of death and disease [43].

Given the importance of space and place to the critical question of who lives and dies, and how we live and die, there are additional questions which must be answered. For example, whom are cities built for? Whose interests are in mind when a particular neighborhood’s physical infrastructure is allowed to age and crumble? And who is involved in such decision-making, which often amounts to the place-making from above? These are questions beyond the scope of our empirical analysis, but they are nevertheless related to the findings we present.

## Figures and Tables

**Table 1:** Descriptive statistics for study variables in the 500 largest U.S. cities, 2015.

Study Variables	Mean	S.D.
Dependent variables (chronic disease)		
Crude diabetes prevalence rate	10.78	4.24
Crude stroke prevalence rate	3.11	1.42
Crude high blood pressure prevalence rate	30.95	8.02
Structural vulnerabilities		
% Black households	19.87%	27.49%
% Households with one or more persons 65 years+	20.35%	10.87%
Housing stock		
Median year unit built	1966	1918
Median home value	$196,400	$197,903
Socioeconomic composition		
Gini index of inequality (household level)	0.421	0.074
% Households with SNAP without children < 18	6.16%	7.28%
% Less than high school degree	8.01%	8.51%
Weight status (1 = overweight/obese)	73%	0.433

Total sample based on 27,024 census tracts.

**Table 2:** Crude chronic disease prevalence regression models in the 500 largest U.S. cities, 2015.

Variables	Diabetes		Stroke		High Blood Pressure	
	*b*	95% CI	*b*	95% CI	*b*	95% CI
Structural vulnerabilities						
% Black households	0.055 **	0.054, 0.057	0.130 **	0.127, 0.133	0.022 **	0.021, 0.023
% Households with persons 65 years+	0.080 **	0.075, 0.082	0.223 **	0.217, 0.230	0.030 **	0.029, 0.032
Housing characteristics						
Median year units built	−0.002 *	0.000, 0.001	0.001	−0.004, 0.04	−0.003 **	−0.003, −0.001
Median home value	−0.001 *	0.000, 0.001	−0.001 **	0.000, 0.011	−0.001 **	0.000, 0.001
Socioeconomic characteristics						
Gini index of inequality	1.41 **	0.898, 1.91	6.66 **	5.66, 7.65	0.964 **	0.795, 1.13
% Receiving SNAP benefits	0.059 **	0.052, 0.065	0.090 **	0.078, 0.103	0.029 **	0.026, 0.031
% < High school education	0.085 **	0.082, 0.089	−0.038 **	−0.04, −0.03	0.010 **	0.009, 0.012
Weight status (1 = overweight/obese)	0.897 **	0.801, 1.01	1.91 **	1.72, 2.11	0.185 **	0.153, 0.217
Constant	5.09	4.86, 5.31	19.7	19.3, 20.3	1.19	1.14, 1.27
*df*	8		8		8	
Adjusted R^2^	0.54		0.51		0.55	

* *p* < 0.05; ** *p* < 0.001.
